# Association of ultrasound-assisted combined with conventional anatomical landmark paramedian spinal anesthesia and its impact on first pass success rate in patients with lower limb fractures- A retrospective cohort study

**DOI:** 10.1371/journal.pone.0334455

**Published:** 2025-10-22

**Authors:** Congli Tian, Jing Xue, Huiyan Cui, Hongni Ding

**Affiliations:** 1 Department of Anesthesia and Surgery, Zhengning County People’s Hospital, Qingyang, Gansu Province, China; 2 Department of Anesthesiology, Qingyang People’s Hospital, Qingyang, Gansu Province, China; 3 Department of Anesthesia and Surgery, Zibo City Centre Hospital, Zibo, Shandong Province, China; 4 Anesthesia and Surgicai Department, Qingyang People’s Hospital, Qingyang, Gansu Province, China; Yarmouk University, JORDAN

## Abstract

**Background:**

The association between ultrasound-assisted combined with conventional anatomical landmark paramedian spinal anesthesia regarding the first-pass success rate remains contentious. This study aims to clarify this relationship.

**Methods:**

In this retrospective cohort analysis of 146 patients with lower limb fractures, patients were divided into two groups based on their spinal anesthesia technique:ultrasound-assisted combined with conventional anatomical landmark (median or paramedian). The primary endpoint was the first-pass success rate, while secondary endpoints included total procedure time and discomfort score. Recorded covariates encompassed sex, age, BMI, ASA class, preoperative preparation time, pre-injury physical activity, number of needle insertions, pain intensity (VAS), satisfaction score, puncture-related pain, and postoperative low back pain. Outcomes were evaluated *via* logistic regression.

**Results:**

After adjusting for potential confounders, the ultrasound-assisted paramedian approach demonstrated a significantly higher first-pass success rate (OR = 6.11, 95% CI 2.09–17.86, P = 0.001). Secondary benefits included reduced procedure duration and improved patient comfort. Sensitivity analysis using propensity score matching confirmed the robustness of the results (E-value = 4.379), indicating minimal influence from unmeasured confounders.

**Conclusions:**

In patients with lower limb fractures, ultrasound-assisted paramedian spinal anesthesia using anatomical landmarks increased first-pass success rates, reduced procedural time, and improved patient comfort compared to the median approach.

## 1 Introduction

Spinal anesthesia are increasingly favored for limb and abdominal surgeries due to their lower cost, technical simplicity, and greater patient acceptance [1,2]. However, success rates differ depending on the technique used [3], and multiple attempts may elevate the risk of complications while diminishing patient satisfaction [4]. In clinical anesthesia, ultrasound assistance, which addresses the limitations of traditional landmark palpation, markedly enhances first-attempt success rates, particularly in complex cases [5,6,7–10]. Neuraxial ultrasound provides precise identification of intervertebral spaces and measures epidural/intrathecal depth [11], enabling less-experienced anesthesiologists to predict challenges and optimize needle placement. Despite ultrasound’s well-established advantages in neuraxial anesthesia [12–17], critical knowledge gaps persist regarding its optimal use for spinal anesthesia in patients with lower limb fractures, particularly in resource-limited primary hospitals. Current literature lacks: (1) standardized ultrasound-assisted protocols for this patient population, (2) comparative data on anatomical approaches, and (3) evidence from primary care settings in developing countries [18]. This study explores whether combining ultrasound guidance with anatomical landmark techniques improves first-attempt success rates of spinal anesthesia in this cohort.

## 2 Methods

### 2.1. Database source and ethical statement

This retrospective study analyzed data from patients aged ≥ 18 years who underwent spinal anesthesia for lower limb fractures at Zhengning County People’s Hospital, China, during the period from October 1, 2024, to April 30, 2025. Data were extracted from structured anesthesia record, including baseline characteristics, surgical details, complications, and results from various assessment scales. Three anesthesiologists performed the ultrasound-assisted combined with conventional anatomical landmark spinal anesthesia technique in accordance with the institutional protocol.Spinal anesthesia was administered in the lateral position using 25G needles at the L3-L4 or L4-L5 interspace. Following confirmation of free cerebrospinal fluid flow, 8–12.5 mg of 0.5% bupivacaine was injected.The anesthesiologist calculates the dosage according to the patient’s weight (0.15 mg/kg), age, complications, and surgical duration. Continuous intravenous access and vital sign monitoring were maintained throughout the procedure.

The study received ethical approval from the Ethics Committee of Zhengning County People’s Hospital, located in Qingyang City, Gansu Province, China, on January 10, 2024 (Approval Number: 202401001). It has also been registered as a management project in Qingyang City (Project Number: QY-STK-2025B-010). All methods employed in this study complied with relevant ethical guidelines and regulations and adhered to established standards for epidemiological observational studies. Since this study used anonymous secondary data without patient intervention, informed consent was waived.

### 2.2. Sample size calculation and Spinal Anesthesia Procedure

Given the 15-month study period, consecutive case selection was employed. Based on Fleiss’ formula (88% exposure vs. 68% non-exposure rate, 20% risk difference; α = 0.05, two-tailed; 80% power; 1:1 allocation), the required sample size was 67 per group (total n = 134). Accounting for attrition, the target was increased to 158. All eligible patients within the database period were included to minimize selection bias.

Spinal anesthesia was administered by an anesthesiologist using an ultrasound-assisted technique combined with conventional anatomical landmarks.The procedure commenced with ultrasound localization in either a transverse or paramedian sagittal plane using a SonoScape Edge ultrasound machine (Model JY-SS-028) equipped with a 3.5–5.0 MHz convex probe. Subsequently, the anesthesiologist performed localization using the conventional Tuffier’s line palpation method. When the ultrasound-assisted localization matched the gap identified by the traditional method, this gap was selected for puncture; in cases of discrepancy, the ultrasound-determined gap was used as the final puncture site.Upon identifying the optimal intervertebral space, a 25-gauge needle (Henan Tuoren Medical Devices Co., Ltd.) was inserted via either the median or paramedian approach. Dural puncture was confirmed by the backflow of cerebrospinal fluid (CSF). If three attempts were unsuccessful, the approach was adjusted (median to paramedian or vice versa) based on decision-making by the same anesthesiologist. General anesthesia was considered when anatomical landmarks between L5-S1 and L3-4 could not be clearly identified with unsuccessful puncture.

### 2.3. Study population

Inclusion criteria: Adult patients (≥ 18 years) undergoing ultrasound-assisted spinal anesthesia for lower limb fracture surgery. Exclusion criteria: (1) Puncture site infection, (2) Pre-existing lower limb neurological deficits, (3) Contraindications to regional anesthesia or local anesthetic allergy, (4) Coagulation disorders.

### 2.4. Main exposure

The primary independent variable was ultrasound-assisted paramedian spinal anesthesia combined with traditional landmark techniques. This approach utilized preprocedural ultrasound guidance (Edge series, SonoSound Medical Equipment Trading [Shanghai], Model JY-SS-028) to identify the puncture site in conjunction with conventional anatomical landmarks.

### 2.5. Covariates

Baseline patient characteristics, including sociodemographics (gender, age, BMI, ASA class, preoperative wait time) and comorbidities (back pain, rheumatoid arthritis, prior anesthesia history, activity level), were collected. Preoperative waiting time was defined as days from hospital admission to surgery.Preoperative imaging was reviewed to classify fracture types, and all procedures were performed by qualified anesthesiologists with more than five years of experience and ultrasound certification. A 48-hour postoperative evaluation was conducted, including: (1) pain intensity (VAS 0–10), (2) procedure-related discomfort (NRS 0–10, evaluating anxiety, fear, and positioning discomfort), and (3) patient satisfaction (NRS 0–10). Complications such as headache, persistent back pain, dysesthesia, and puncture-induced numbness (defined as tingling or electric shock sensations radiating from the lumbar region to the thighs) were also recorded.

### 2.6. Outcome measures

The primary outcome was the First Pass Success Rate, defined as successful cerebrospinal fluid aspiration with a single needle insertion, without redirection or reinsertion. Secondary outcomes included: the number of attempts (total needle advancements, including withdrawals and redirections from the initial puncture), discomfort score (measured on a 10-point NRS, where 0 represents no discomfort and 10 represents maximum discomfort, incorporating anxiety, fear, and positioning-related discomfort), and total procedure time (from landmark identification to completion of anesthesia administration).

### 2.7. Statistical analysis

The histogram distribution and the Kolmogorov-Smirnov test were used to assess the normality of variable distributions. Continuous variables with normal distributions are presented as mean ± standard deviation (SD), while those with skewed distributions are reported as median (interquartile range [IQR]). Categorical variables are expressed as frequencies (%). Differences between paired factors within groups were analyzed using the paired t-test or the Wilcoxon signed-rank test. For comparisons of continuous variables across groups, either the independent samples Student’s t-test or the Mann-Whitney U-test was applied, depending on the distribution of the data. Comparisons of categorical data were conducted using the chi-square test or Fisher’s exact test, as appropriate.

Odds ratios (OR) and 95% confidence intervals were estimated to assess the association between the ultrasound-assisted combined traditional positioning paramedian group and first-pass success. Conditional logistic regression models were employed for matched sets. Confounder selection was guided by clinical relevance, existing literature, significant covariates identified in univariate analysis, and any changes in effect estimates greater than 10%. Five models were developed for analysis. Model 1 was adjusted for sex, age, BMI, ASA classification, and preoperative waiting time. Model 2 additionally accounted for lower back pain, rheumatoid arthritis, surgical anesthesia history, and physical activity. Model 3 incorporated the VAS score, NRS comfort score, satisfaction, needle sensation, postoperative low back pain, and postoperative headache. Model 4 included adjustments for fracture type and anesthesiologist. Model 5, our primary model, included all the aforementioned variables.

To minimize potential biases and confounding factors in treatment allocation, propensity score matching (PSM) [19] was used to estimate the likelihood of successful first puncture in the ultrasound-assisted combined traditional paramedian group. A 1:1 nearest neighbor matching algorithm with a matching width of 0.2 was applied, with covariates such as gender, age, BMI, ASA classification, preoperative waiting time, low back pain, rheumatoid arthritis, surgical anesthesia history, physical activity, VAS score, NRS comfort score, satisfaction, puncture discomfort, postoperative low back pain, postoperative headache, fracture type, and anesthesiologist adjustments used to generate propensity scores. The effectiveness of PSM was evaluated using standardized mean differences (SMD), with values below 0.1 considered acceptable.

Subgroup and secondary outcome analyses were conducted for further exploration. Continuous variables were categorized using clinical cut points or tertiles before performing interaction tests. Missing data, which constituted less than 5% of the dataset, were addressed through listwise deletion. Sensitivity analyses were performed to evaluate the robustness of the findings and the influence of different association models on the conclusions. Various models, including propensity score adjusted (PSA) [20], PSM [21], inverse probability of treatment weighting (IPTW) [22], pairwise algorithmic (PA) [23], and overlap weight (OW) [24], were employed. Effect sizes and p-values were calculated, reported, and compared across all models. Additionally, the e-value was computed to assess the potential impact of unmeasured confounders on the relationship between the ultrasound-assisted combined traditional positioning paramedian group and first-pass success.

All analyses were performed using R Statistical Software (Version 4.2.2, http://www.R-project.org, The R Foundation) and the Free Statistics analysis platform (Version 1.9, Beijing, China, http://www.clinicalscientists.cn/freestatistics). A two-sided P-value of less than 0.05 was considered statistically significant.

## 3 Results

### 3.1 Study population

**Fig 1:**
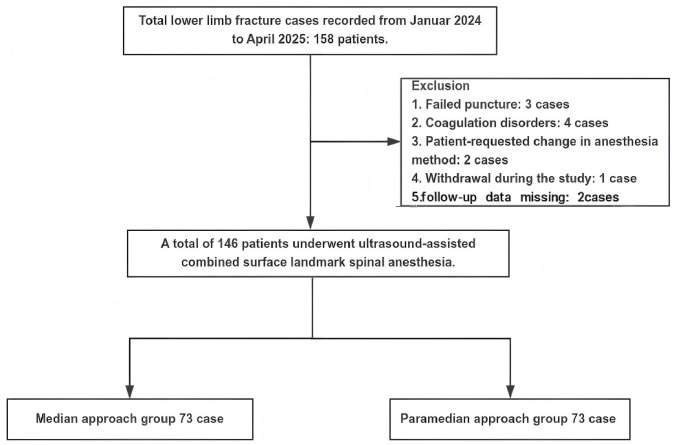
Flow chart of this study. This study initially enrolled 158 patients scheduled for lower limb fracture surgery. Twelve were excluded: three due to failed punctures, four for coagulopathy, two who requested a different anesthesia method, one who withdrew, and two for incomplete follow-up data. Ultimately, 146 patients underwent ultrasound-assisted combined traditional spinal anesthesia, categorized into median and paramedian approaches.

This study initially enrolled 158 patients undergoing lower limb fracture surgery. After excluding 12 patients (3 failed punctures, 4 coagulation disorders, 2 anesthesia preference changes, 1 withdrawal, and 2 follow-up data missing), 146 patients received ultrasound-assisted spinal anesthesia. These patients were subsequently categorized into two groups based on their spinal anesthesia approach using an ultrasound-assisted technique combined with conventional anatomical landmarks (median or paramedian). (Fig 1).

### 3.2 Baseline characteristics

The baseline characteristics of the patients are presented in Table 1.The mean age of participants was 61.6 years, with 79 (54.1%) women and 67 (45.9%) men.Among all patients, 107 (73.3%) were ASA I-II and 39 (26.7%) were ASA III.The mean duration of preoperative preparation (hospital admission to surgery) was 6.3 days. The first puncture success rate was 54.8% (40/73) in the median approach group and 82.2% (60/73) in the paramedian approach group.

**Table 1:** Baseline characteristics of patients by Puncture Method Groups.

Variables	Total (n = 146)	Median group (n = 73)	Paramedian group (n = 73)	P-value
**Sex n(%)**				0.245
**Male**	67 (45.9)	37 (50.7)	30 (41.1)	
**Female**	79 (54.1)	36 (49.3)	43 (58.9)	
**Age(years), Mean ± SD**	61.6 ± 14.6	58.7 ± 14.5	64.6 ± 14.1	0.014
**BMI(kg/m** ^**2**^ **),Mean ± SD**	22.1 ± 3.3	22.0 ± 3.0	22.2 ± 3.6	0.713
**ASA, n (%)**				0.19
**I, II**	107 (73.3)	57 (78.1)	50 (68.5)	
**III**	39 (26.7)	16 (21.9)	23 (31.5)	
**Waiting time for surgery(day),Mean ± SD**	6.3 ± 3.4	7.0 ± 3.8	5.6 ± 2.9	0.015
**Comorbidities**				
**Lower back pain, n (%)**				1
**No**	130 (89.0)	65 (89)	65 (89)	
**Yes**	16 (11.0)	8 (11)	8 (11)	
**Rheumatoid arthritis, n (%)**				0.209
**No**	140 (95.9)	72 (98.6)	68 (93.2)	
**Yes**	6 (4.1)	1 (1.4)	5 (6.8)	
**History of surgical anesthesia, n (%)**				0.249
**No**	110 (75.3)	58 (79.5)	52 (71.2)	
**Yes**	36 (24.7)	15 (20.5)	21 (28.8)	
**Physical activity before injury, n (%)**				0.224
**Mild-Moderate**	95 (65.1)	44 (60.3)	51 (69.9)	
**severe**	51 (34.9)	29 (39.7)	22 (30.1)	
**The first puncture was successful, n (%)**				< 0.001
**No**	46 (31.5)	33 (45.2)	13 (17.8)	
**Yes**	100 (68.5)	40 (54.8)	60 (82.2)	
**Number of puncture attempts, n (%)**				0.085
**First time**	87 (59.6)	37 (50.7)	50 (68.5)	
**second time**	24 (16.4)	14 (19.2)	10 (13.7)	
**third time**	35 (24.0)	22 (30.1)	13 (17.8)	
**Success on the first attempt, n (%)**				0.017
**No**	56 (38.4)	35 (47.9)	21 (28.8)	
**Yes**	90 (61.6)	38 (52.1)	52 (71.2)	
**Degree of satisfaction, n (%)**				1
**No**	44 (30.1)	22 (30.1)	22 (30.1)	
**Yes**	102 (69.9)	51 (69.9)	51 (69.9)	
**Needle sensation, n (%)**				0.064
**No**	124 (84.9)	66 (90.4)	58 (79.5)	
**Yes**	22 (15.1)	7 (9.6)	15 (20.5)	
**Postoperative low back pain, n (%)**				0.701
**No**	110 (75.3)	54 (74)	56 (76.7)	
**Yes**	36 (24.7)	19 (26)	17 (23.3)	
**Postoperative headache, n (%)**				0.512
**No**	136 (93.2)	67 (91.8)	69 (94.5)	
**Yes**	10 (6.8)	6 (8.2)	4 (5.5)	
**Fracture type, n (%)**				0.244
**Distal lower limb**	65 (44.5)	36 (49.3)	29 (39.7)	
**Proximal lower limb**	81 (55.5)	37 (50.7)	44 (60.3)	
**Anesthesiologist, n (%)**				0.174
**A**	36 (24.7)	20 (27.4)	16 (21.9)	
**B**	42 (28.8)	17 (23.3)	25 (34.2)	
**C**	31 (21.2)	13 (17.8)	18 (24.7)	
**D**	37 (25.3)	23 (31.5)	14 (19.2)	
**Execution time(s), Median (IQR)**	60.0 (31.5, 135.0)	80.0 (60.0, 180.0)	60.0 (23.0, 120.0)	0.001
**Total operation time(s), Median (IQR)**	180.0 (110.0, 300.0)	180.0 (120.0, 320.0)	150.0 (80.0, 240.0)	0.02
**VAS score, Median (IQR)**	1.0 (0.0, 2.0)	1.0 (0.0, 3.0)	1.0 (0.0, 2.0)	0.407
**NRS Comfort rating score, Median (IQR)**	1.0 (0.0, 2.0)	1.0 (0.0, 3.0)	1.0 (0.0, 2.0)	0.688

**BMI:Body Mass Index;ASA:American Society of Anesthesiologists;VAS:Visual Analog Scale;NRS:Numerical Rating Scale.**

### 3.3 Association between covariates and first pass success rate

In univariate analysis, the paramedian approach group demonstrated shorter anesthesia(COR = 0.98, 95% CI = 0.97–0.99, P < 0.001) and operation times(COR = 0.99, 95% CI = 0.99–0.99, P < 0.001), a higher first-pass success rate(COR = 3.81, 95% CI = 1.79–8.11, P = 0.001), and lower pain scores(COR = 0.79, 95% CI = 0.68–0.91, P = 0.002), resulting in a better overall patient experience compared to the median approach group. Age showed a statistically significant difference (Table 2).

**Table 2:** Associations between covariates and the first pass was successful.

Variable	COR_95 CI	P_value	Variable	COR_95 CI	P_value
**Sex**			**ASA**		
**Male**	1(reference)		**Ⅰ,Ⅱ**	1(reference)	
**Fmale**	0.99 (0.49 ~ 1.99)	0.969	**Ⅲ**	0.56 (0.26 ~ 1.2)	0.137
**Age(years)**	0.97 (0.95 ~ 1)	0.046	**BMI(kg/m2)**	0.95 (0.86 ~ 1.06)	0.386
**Execution time(s)**	0.98 (0.97 ~ 0.99)	<0.001	**Total operation time(s)**	0.99 (0.99 ~ 0.99)	<0.001
**Puncture method**			**Physical activity**		
**Medlian group**	1(reference)		**Mild-Moderate**	1(reference)	
**Paramedian group**	3.81 (1.79 ~ 8.11)	0.001	**Severe**	1.56 (0.73 ~ 3.32)	0.253
**Lower back pain**			**Rheumatoid arthritis**		
**No**	1(reference)		**No**	1(reference)	
**Yes**	2.14 (0.58 ~ 7.92)	0.254	**Yes**	7659176.56 (0 ~ Inf)	0.987
**Success on the first attempt**			**degree of satisfaction**		
**No**	1(reference)		**No**	1(reference)	
**Yes**	95.92 (25.95 ~ 354.62)	<0.001	**Yes**	1.58 (0.75 ~ 3.33)	0.225
**History of surgical anesthesia**			**Fracture type**		
**No**	1(reference)		**Distal lower limb**	1(reference)	
**Yes**	0.89 (0.4 ~ 2)	0.786	**Proximal lower limb**	0.48 (0.23 ~ 1)	0.051
**Needle sensation**			**Postoperative low back pain**		
**No**	1(reference)		**No**	1(reference)	
**Yes**	1.27 (0.46 ~ 3.49)	0.643	**Yes**	0.76 (0.34 ~ 1.68)	0.494
**Waiting time**	0.98 (0.89 ~ 1.09)	0.739	**Number of puncture attempts**		
**for surgery(day)**			**First time**	1(reference)	
**VAS score**	0.79 (0.68 ~ 0.91)	0.002	**Second time**	0.01 (0 ~ 0.06)	<0.001
**NRS Comfort rating score**	0.76 (0.65 ~ 0.88)	<0.001	**Third time**	0 (0 ~ 0.02)	<0.001
**Postoperative headache**			**Anesthesiologist**		
**No**	1(reference)		**A**	1(reference)	
			**B**	1.41 (0.52 ~ 3.84)	0.504
**Yes**	1.08 (0.27 ~ 4.37)	0.915	**C**	0.8 (0.29 ~ 2.22)	0.669
			**D**	0.72 (0.27 ~ 1.91)	0.513

**BMI:Body Mass Index;ASA:American Society of Anesthesiologists;VAS:Visual Analog Scale;NRS:Numerical Rating Scale.**

### 3.4 Association between ultrasound-assisted traditional anatomical techniques and first pass success rate in spinal anesthesia

In a multivariate analysis of ultrasound-assisted spinal anesthesia, logistic regression indicated that participants using the paramedian approach had a first-pass success rate 3.81 times greater than those using the median approach (COR = 3.81, 95% CI = 1.79–8.11, P = 0.001). This association remained statistically significant after adjusting for confounding variables, including gender, age, BMI, ASA classification, preoperative waiting time, history of lower back pain, rheumatoid arthritis, prior surgical anesthesia, physical activity levels, VAS score, NRS comfort score, satisfaction levels, acupuncture sensation, postoperative lower back pain, postoperative headache, fracture type, and anesthetist (AOR = 6.11, 95% CI = 2.09–17.86, P = 0.001), as shown in Table 3.

**Table 3:** Adjusted odds ratios for risk of the first pass was successful.

Puncture method	n total	n event_%	Crude Model		Model-Ⅰ		Model-Ⅱ		Model-Ⅲ		Model-IV	
			COR_95 CI	P value	AOR_95 CI	P value	AOR_95 CI	P value	AOR_95 CI	P value	AOR_95 CI	P value
**Median group**	73	40 (54.8)	1(Ref)		1(Ref)		1(Ref)		1(Ref)		1(Ref)	
**Paramedian group**	73	60 (82.2)	3.81 (1.79 ~ 8.11)	0.001	6.41 (2.62 ~ 15.7)	<0.001	5.98 (2.43 ~ 14.72)	<0.001	5.71 (2.08 ~ 15.7)	0.001	6.11 (2.09 ~ 17.86)	0.001

OR, odds ratio; CI, confifidence interval; Ref: reference

Crude model unadjusted for model

Model 1 was adjusted for sociodemographic variables (sex,age,BMI, ASA classification, and preoperative waiting time).

Model 2 adjusted for the variables in model 1 plus lower back pain, rheumatoid arthritis;history of surgical anesthesia;physical activity.

Model 3 adjusted for the variables in model 2 plusVAS score;NRS comfort rating score;degree of satisfaction;needle sensation;postoperative low back pain;postoperative headache.

Model 4 adjusted for the variables in model 3 plus fracture type and anesthesiologist.

### 3.5 Sensitive analysis

**Fig 2:**
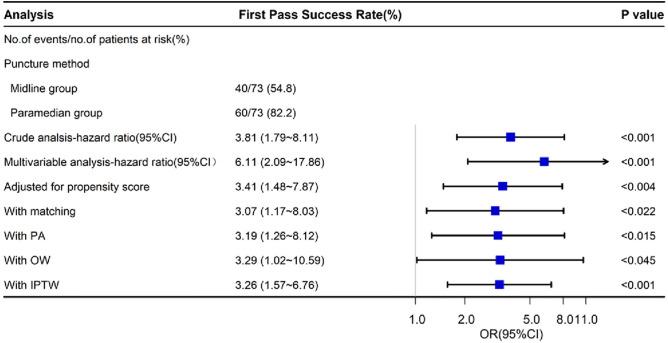
Forest plot illustrating the odds ratios (ORs) for the association between ultrasound-assisted combined conventional paramedian spinal anesthesia and first-pass success rate.

A sensitivity analysis was performed to assess the robustness of the findings and the impact of various association models on the results. The analysis employed PA, OW, and IPTW. The paramedian approach was associated with an increased first-pass success rate, yielding a PA OR of 3.19 (95% CI 1.26 to 8.12, p < 0.015), an OW OR of 3.29 (95% CI 1.02 to 10.59, p < 0.045), and an IPTW OR of 3.26 (95% CI 1.57 to 6.76, p < 0.001) (Fig 2). Furthermore, the E-value ranged from 4.379 to 2.248 for this cohort (Table 4).

**Table 4:** The E value range of the queue.

Item	point	lower	upper
**AOR**	2.472	1.446	4.226
**E-values**	4.379	2.248	

### 3.6 Secondary outcomes

After adjusting for confounding factors, secondary outcome analysis demonstrated that ultrasound-assisted combined with conventional anatomical landmark paramedian spinal anesthesia reduced the number of puncture attempts and total procedure time compared to the median approach, while also improving patient comfort (Table 5).

**Table 5:** Secondary outcome analyses.

variable	Median		Paramedian	variable	Median		Paramedian
n.total	73		73	n.total	73		73
n.event_%	40 (54.8)		60 (82.2)	n.event_%	40 (54.8)		60 (82.2)
		β_95 CI	P-value			AOR_95 CI	P-value
Abstract needle attempts	1(Ref)	−0.7(−1.28 ~ −0.12)	0.019	satisfaction ratings	1(Ref)	0.89(0.39 ~ 2.02)	0.785
total surgery time	1(Ref)	−105.62(−183.22 ~ −28.01)	0.009	Piercing sensation	1(Ref)	2.55(0.86 ~ 7.54)	0.09
pain intensity	1(Ref)	−0.56(−1.37 ~ 0.25)	0.177	Lower back pain	1(Ref)	0.62(0.26 ~ 1.47)	0.275
discomfort scores	1(Ref)	−0.92 (−1.77 ~ −0.06)	0.037				

OR, odds ratio; Beta coefficient;CI, confifidence interval; Ref: reference.

The analysis was adjusted for sex, age, BMI, ASA classification, preoperative waiting time, lower back pain, rheumatoid arthritis, history of spinal anesthesia, physical activity, VAS score, NRS comfort rating score, satisfaction level, needle sensation, postoperative low back pain, postoperative headache, fracture type, and anesthesiologist.

### 3.7 Subgroup analyses

**Fig 3:**
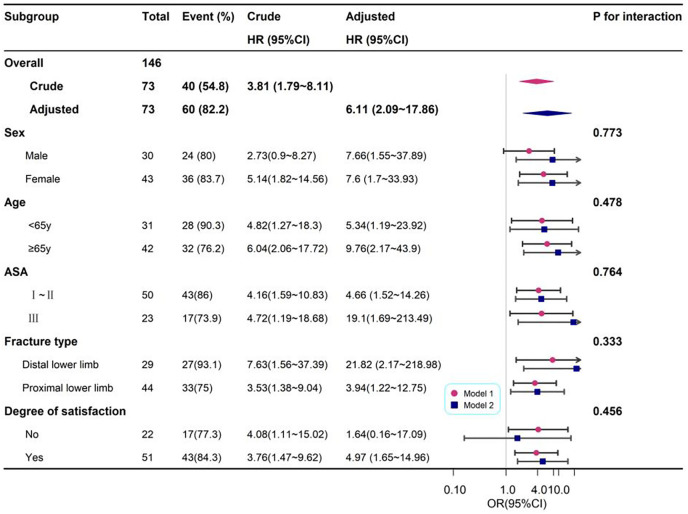
Stratified analyses based on additional variables.

Subgroup analyses were performed based on gender, age, ASA classification, physical activity, type of surgery, and satisfaction scores. Forest plots illustrated both unadjusted and adjusted OR. All covariates, except for the stratification variable, were adjusted. No significant interactions were observed within the subgroups (Fig 3).

## 4 Discussion

The study demonstrates that ultrasound-assisted paramedian spinal anesthesia, guided by anatomical landmarks, significantly increases the first-pass success rate. Additionally, it reduces the number of attempts, shortens procedure time, and improves patient comfort. These findings are crucial for improving success rates in Class II Grade A hospitals in China, where such techniques are underused.

Supporting evidence from previous studies aligns with these results [25,26,27,28]. Ultrasound assistance has been shown to improve first-pass success in spinal anesthesia for lower extremity fractures. The underlying mechanisms can be explained through five interrelated pathways, reinforcing existing literature while offering new clinical insights. Ultrasound outperforms palpation methods, particularly in challenging anatomical cases, as evidenced by an 85.7% (P < 0.038) first-attempt success rate in obese patients (Jain K et al. [9,15,29]). Real-time ultrasound visualization of the lamina and interlaminar space optimizes needle trajectory, reducing bone contact attempts by 43% compared to the median approach, consistent with Khan MA et al.‘s findings on first-puncture success [25,30]. The ultrasound-assisted paracentral approach allows for real-time angle adjustments during needle advancement, minimizing multi-plane redirection attempts and saving time [28]. High-frequency ultrasound can differentiate ligamentous layers, especially the ligamentum flavum-dura mater complex, providing objective identification of anatomical boundaries. This mechanical advantage was demonstrated in Chong SE et al.’s study [14,31]. Moreover, ultrasound-assisted observation of cerebrospinal fluid flow and anesthetic spread, in conjunction with anatomical palpation, offers a transformative safety approach, consistent with pooled evidence [28,32].

Ultrasound-assisted spinal anesthesia has well-established benefits, though its effectiveness is influenced by factors such as image quality, patient anatomy, and operator experience. Both transverse median (TM) and parasagittal oblique (PSO) approaches are particularly prone to image degradation due to lumbar degeneration [32]. Our results confirm the 67.1% first-attempt success rate reported in the 2023 meta-analysis [33], while significantly extending clinical applicability through paramedian techniques. This study diverges from previous research in three key areas [34–36]: first, it demonstrates consistent efficacy across all adult age groups (18–86 years) without the age-related limitations observed in earlier subgroup analyses (elderly patients >65 years); second, it documents improved patient outcomes through fewer attempts and reduced procedure times; and third, it validates the implementation of ultrasound-assisted techniques in resource-limited settings, specifically in China’s Class II Grade A hospitals, where ultrasound remains underutilized. The effectiveness of non-real-time ultrasound assistance suggests that simplified training could enhance accessibility, complementing European guidelines that emphasize ultrasound’s preoperative anatomical mapping value [37]. Our data, however, confirm its intraoperative utility—findings that collectively support broader integration of point-of-care ultrasound (POCUS) in perioperative care [38]. Clinically, ultrasound-assisted paramedian techniques emerge as a practical primary approach, particularly in settings where real-time systems are unavailable, thus overcoming workflow barriers identified in earlier studies [39]. These conclusions align with Chen et al.’s demonstration of superior outcomes with ultrasound-assisted techniques (USAS) compared to real-time guidance (USRTG) in elderly patients with hip fracture (n = 114), regarding success rates, procedural efficiency, and patient satisfaction [39]. The present study, however, extends these advantages across all age groups and fracture types. While the lack of significant differences in complication rates between approaches requires careful interpretation due to potential confounding variables, the divergence from Chen et al.’s age-related findings likely reflects methodological or population differences, highlighting the need for standardized protocols.

The strengths of this study include its large sample size and comprehensive design, which provide robust statistical power to evaluate the impact of ultrasound-assisted combined with conventional landmark techniques (median and paramedian approaches) on first-pass success rates in spinal anesthesia. Our findings offer novel clinical insights with substantial real-world applicability. Using IPTW on data from patients with lower limb fractures, this study assessed whether ultrasound-assisted anatomical localization improved first-attempt success rates in the paramedian approach group. Rigorous adjustments for confounders and biases further strengthen the evidence supporting this correlation.

Nonetheless, several limitations warrant consideration. First, the retrospective design precluded prospective sample size calculation, although post-hoc power analyses were performed and reported. Second, heterogeneity in study designs and outcome measures complicates the mechanistic interpretation of the results. Third, the short follow-up period limited the detection of clinical events, particularly reducing statistical power for assessing postoperative low back pain and long-term complications. Fourth, the lack of standardized ultrasound image quality assessment may have affected technical difficulty, especially for median approaches. Although multivariate and subgroup analyses demonstrated consistent results after covariate adjustment, the single-center design of this Chinese cohort requires external validation across diverse populations. Despite these constraints, this study provides a comprehensive evaluation of ultrasound-assisted spinal anesthesia techniques.

## 5 Conclusion

This study demonstrates that ultrasound-assisted paramedian spinal anesthesia, utilizing anatomical landmarks, significantly improves first-pass success rates in patients with lower limb fractures. This combined approach reduces procedure time, minimizes puncture attempts, and improves patient comfort. Future research should explore long-term outcomes and evaluate the broader applicability of these techniques across diverse patient populations.

## Supporting information

S1 Data (CSV)

## Abbreviations

BMI: Body Mass Index
ASA: American Society of Anesthesiologists
VAS: Visual Analog Scale
NRS: Numerical Rating Scale
POCUS: Point-of-Care Ultrasound
USAS: Ultrasound-Assisted
USRTG: Ultrasound Real-Time Guidance
